# Chylomicrons stimulate incretin secretion in mouse and human cells

**DOI:** 10.1007/s00125-017-4420-2

**Published:** 2017-09-02

**Authors:** Arianna Psichas, Pierre F. Larraufie, Deborah A. Goldspink, Fiona M. Gribble, Frank Reimann

**Affiliations:** Metabolic Research Laboratories and MRC Metabolic Diseases Unit, WT-MRC Institute of Metabolic Science, Addenbrooke’s Hospital, University of Cambridge, Cambridge, CB2 0QQ UK

**Keywords:** Chylomicrons, Enteroendocrine, Fat-sensing, *Ffar1*/GPR40, Glucagon-like peptide-1, Glucose-dependent insulinotropic peptide, GPR119, Gut peptide, Incretin, Lipoprotein lipase

## Abstract

**Aims/hypothesis:**

Lipids are a potent stimulus for the secretion of glucagon-like peptide (GLP)-1 and glucose-dependent insulinotropic peptide (GIP). Traditionally, this effect was thought to involve the sensing of lipid digestion products by free fatty acid receptor 1 (FFA1) and G-protein coupled receptor 119 (GPR119) on the apical surface of enteroendocrine cells. However, recent evidence suggests that lipids may in fact be sensed basolaterally, and that fatty acid absorption and chylomicron synthesis may be a prerequisite for their stimulatory effect on gut peptide release. Therefore, we investigated the effect of chylomicrons on GLP-1 and GIP secretion in vitro.

**Methods:**

The effect of chylomicrons on incretin secretion was investigated using GLUTag cells and duodenal cultures of both murine and human origin. The role of lipoprotein lipase (LPL) and FFA1 in GLUTag cells was assessed by pharmacological inhibition and small (short) interfering RNA (siRNA)-mediated knockdown. The effect of chylomicrons on intracellular calcium concentration ([Ca^2+^]_i_) was determined by imaging GLUTag cells loaded with Fura-2. In the primary setting, the contributions of FFA1 and GPR119 were investigated using L cell-specific *Gpr119* knockout cultures treated with the FFA1 antagonist GW1100.

**Results:**

Chylomicrons stimulated GLP-1 release from GLUTag cells, and both GLP-1 and GIP secretion from human and murine duodenal cultures. Chylomicron-triggered GLP-1 secretion from GLUTag cells was largely abolished following lipase inhibition with orlistat or siRNA-mediated knockdown of *Lpl*. In GLUTag cells, both GW1100 and siRNA-mediated *Ffar1* knockdown reduced GLP-1 secretion in response to chylomicrons, and, consistent with FFA1 G_q_-coupling, chylomicrons triggered an increase in [Ca^2+^]_i_. However, LPL and FFA1 inhibition had no significant effect on chylomicron-mediated incretin secretion in murine cultures. Furthermore, the loss of GPR119 had no impact on GLP-1 secretion in response to chylomicrons, even in the presence of GW1100.

**Conclusions/interpretation:**

Chylomicrons stimulate incretin hormone secretion from GLUTag cells as well as from human and murine duodenal cultures. In GLUTag cells, the molecular pathway was found to involve LPL-mediated lipolysis, leading to the release of lipid species that activated FFA1 and elevated intracellular calcium.

**Electronic supplementary material:**

The online version of this article (10.1007/s00125-017-4420-2) contains peer-reviewed but unedited supplementary material, which is available to authorised users.

## Introduction

The upper small intestine harbours a rich population of enteroendocrine cells (EECs) [1], which release gut peptides following the ingestion of a meal. Lipids are an established potent stimulus for the secretion of gut peptides including incretins, glucagon-like peptide (GLP)-1 and glucose-dependent insulinotropic peptide (GIP) [2–4]. These peptide hormones serve to facilitate the efficient digestion and absorption of lipids and other nutrients, and to promote satiety. However, despite significant advances in the field of intestinal nutrient sensing in recent years, there are still gaps in our understanding of the mechanisms underlying this fundamental component of postprandial physiology.

Triacylglycerols are digested by lipases in the small intestinal lumen to generate long-chain fatty acids (LCFAs) and 2-monoacylglycerol. These digestion products mediate the stimulatory effect of lipids on gut peptide secretion, acting via G-protein-coupled receptors (GPCRs) expressed on EECs [5–7]. LCFAs are well-established agonists of the free fatty acid receptors 1 and 4 (FFA1 [GPR40] and FFA4 [GPR120]) [8–10]. Until recently, activation of these receptors was thought to trigger gut peptide release solely via activation of G_q_-coupled intracellular pathways, as is indeed the case for the native ligands, long-chain non-esterified fatty acids (NEFAs). However, a new generation of synthetic ‘full’ FFA1 agonists appears able to activate G_s_-coupled signalling in addition to G_q_, and consequently yield greater secretory responses [11]. Monoacylglycerols on the other hand, in particular 2-oleyolglycerol, are known to induce GLP-1 secretion by activating the G_s_-coupled GPR119 receptor [12–14]. Secondary messages downstream of GPCRs thus include elevation of cytosolic Ca^2+^ and cAMP concentrations as well as activation of protein kinases such as extracellular signal-regulated kinase (ERK) [15].

Lipid-sensing receptors have traditionally been thought to be expressed on the apical side of EECs and therefore, in the ‘classical’ model of intestinal fat-sensing, it is luminal lipid digestion products that are responsible for receptor activation. However, recent evidence has called into question the presence of FFA1 at least on the apical surface of EECs. Ex vivo studies using isolated perfused rat intestine revealed that linoleic acid and four synthetic FFA1 agonists elicited GLP-1 secretion only when administered into the vasculature, and not when administered directly into the gut lumen [16]. These findings raise the possibility that FFA1 may actually reside on the basolateral surface of EECs, and that fatty acid absorption may be a prerequisite for lipid stimulated gut peptide release.

Following the absorption of LCFAs and 2-monoacylglycerol by enterocytes, these are re-esterified into triacylglycerols and packaged into chylomicrons. Chylomicrons contain a central triacylglycerol-rich lipid core (approximately 95% of the particle mass) and an outer layer composed of phospholipids, apolipoproteins and non-esterified cholesterol [17]. Mature chylomicrons are then trafficked in vesicles to the basolateral surface of enterocytes, from where they are released. At this point, chylomicrons could interact with the basolateral surface of EECs. Indeed, there is in vivo evidence in support of a role for chylomicron formation and secretion in lipid-induced GLP-1 and GIP secretion. When chylomicron formation was inhibited using the surfactant Pluronic L81, the GLP-1 response to an intraduodenal lipid emulsion in rats was significantly reduced and the increase in GIP was practically abolished [18].

Therefore, we investigated the effect of chylomicrons on GLP-1 and GIP secretion in vitro using GLUTag cells, an established L cell model, as well as primary murine and human duodenal cultures. The mechanisms underlying this effect were explored by employing a combination of small (short) interfering RNA (siRNA)-mediated genetic manipulation and pharmacological inhibition.

## Methods

### Experimental animals and ethical approval

All animal procedures were approved by the University of Cambridge Animal Welfare and Ethical Review Body and conformed to the Animals (Scientific Procedures) Act 1986 Amendment Regulations (SI 2012/3039). The work was performed under the UK Home Office Project License 70/7824. Conditional *Gpr119* knockout mice were generated by crossing homozygous *Gpr119* floxed mice with heterozygous GLUCre12 mice, which express *Cre* recombinase under the control of the proglucagon promoter, as previously described [13]. All mice were on a C57BL/6 background and bred in house. For secretion experiments, intestinal tissue was obtained from both male and female mice on a C57BL6 background (aged 3–8 months). The mice were housed in individually ventilated cages on a 12 h light/dark cycle with ad libitum access to water and regular chow. Mice were culled by approved schedule 1 methods.

### RNA sequencing

High-quality total RNA extracted from approximately 10,000–12,000 FACS-purified L cells and 20,000 non-L cells from the upper small intestine (top 10 cm) of GLU-Venus mice [19] and 50,000 GLUTag cells (a gift from D. Drucker, Lunenfeld-Tanenbaum Research Institute, Toronto, ON, Canada) was used for sequencing, as previously described [20]. Briefly, amplified cDNA was obtained using an Ovation RNA-Seq System V2 (NuGEN, Leek, the Netherlands) and used to generate barcoded libraries (Ovation rapid DR Multiplex System 1-96; NuGEN) after fragmentation to 200 bp. Libraries were SE50 sequenced using an Illumina HiSeq 2500 system (Great Chesterford, UK). After alignment to the mouse genome (GRCm38, https://www.ncbi.nlm.nih.gov/grc/mouse), expression of genes of interest in each sorted population (two positive ‘L cell’ and two negative ‘non-L’ cell populations, and three GLUTag passages) was determined using Cufflinks version 2.2.1 (http://cole-trapnell-lab.github.io/cufflinks/) and expressed as fragments per kilobase per million reads (FPKM).

### Primary intestinal cultures

#### Murine

Duodenal crypts were isolated and cultured as previously described [21] and recently demonstrated [22]. Briefly, the duodenum (top 10 cm distal to the pylorus) was cleaned thoroughly with ice-cold PBS and the muscle layer was removed. The duodenum was cut open longitudinally and minced using a surgical blade to produce tissue pieces of around 1–2 mm^2^. The tissue pieces were then digested with collagenase type XI (0.3 mg/ml high-glucose DMEM) at 37°C and filtered through a 100 μm cell strainer. The resulting cell suspension was plated in a random order onto 24-well plates coated with 2% Matrigel (BD Bioscience, Oxford, UK) for the secretion experiments. The Rho-associated coiled-coil containing protein kinase (ROCK) inhibitor Y-27632 dihydrochloride (Tocris Bioscience, Bristol, UK) was added to final cell suspensions at a concentration of 10 μmol/l to prevent anoikis.

#### Human

The use of human intestinal tissue was approved by the Cambridge Central Research Ethics Committee under license number 09/H0308/24. Fresh surgical specimens of healthy human duodenum were obtained from the Tissue Bank at Addenbrooke’s Hospital (Cambridge, UK). The tissue was stored in L-15 (Leibovitz) medium (Sigma-Aldrich, Poole, UK) at 4°C until processing (within a few hours of surgery). The isolation and culture of mixed human duodenal cells was performed using the same protocol as for murine intestinal cultures, without the filtration step.

### GLUTag cell culture

GLUTag cells, an established pro-glucagon-derived peptide-secreting cell line model [23], were cultured as previously described [24]. Cells for experimental use were plated onto 1% Matrigel-coated (BD BioScience) 24-well plates (secretion) or 35 mm glass-bottomed plastic dishes (MatTek Corporation, Ashland, MA, USA) (imaging). Secretion experiments were carried out 18–24 h after plating. Imaging experiments were carried out 18–48 h after plating.

### siRNA knockdown

GLUTag cells in 24-well plates were transfected with 30 nmol/l AllStars negative control siRNA, *Ffar1* siRNA (Mm_Gpr40_2, target sequence: 5′-TGCGCTGGGCTTTCCATTGAA-3′) or *Lpl* siRNA (Mm_Lpl_5, target sequence: 5′-CAGCTCTATCTTGTTAGTTAA-3′) (Qiagen, Manchester, UK) using Lipofectamine 2000 (Thermo Fisher Scientific, Loughborough, UK) as per the manufacturer’s protocol. Secretion experiments and RNA extraction (wells transfected in parallel) were performed 48 h post-transfection. Knockdown efficiency was assessed using TaqMan gene expression assays (see below).

### Quantitative RT-PCR

Extraction of RNA from siRNA-treated GLUTag cells was performed using an RNeasy Micro Kit (Qiagen). RNA (500 ng) was reverse-transcribed using SuperScript II (Thermo Fisher Scientific), and the resulting cDNA template was mixed with PCR Master Mix (Thermo Fisher Scientific), RNase-free water and specific TaqMan primers (Thermo Fisher Scientific). All experiments were performed on isolated cDNA samples from three independent transfection experiments. In all cases, expression was compared with that of β-actin measured from the same sample in parallel on the same plate, giving a C_t_ difference (ΔC_t_) for β-actin (*Actb*) minus the test gene. Expression is calculated as ${2}^{\Delta {\mathrm{C}}_{\mathrm{t}}}$. The following primer pairs were used (Thermo Fisher Scientific): *Actb*, Mm02619580; *Lpl*, Mm00434764_m1; *Ffar1*, Mm00809442_s1.

### Ca^2+^ imaging

**Fig. 1:**
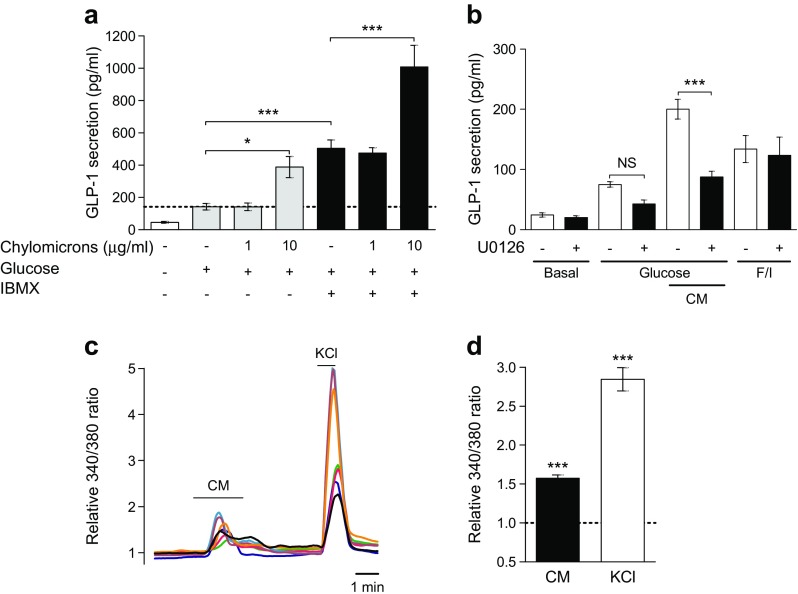
Chylomicrons stimulate GLP-1 secretion in GLUTag cells via an increase in [Ca^2+^]_i_ and activation of ERK signalling. (**a**) GLP-1 secretion from GLUTag cells treated with chylomicrons in the presence of glucose (10 mmol/l) with or without IBMX (100 μmol/l). Data represent means ± SEM, *n* = 12–14 wells from four independent experiments; one-way ANOVA, *p < 0.05, ***p < 0.001. (**b**) GLP-1 secretion from GLUTag cells treated with glucose (10 mmol/l), chylomicrons (CM; 10 μg/ml) or forskolin/IBMX (F/I; 10 μmol/l each) in the presence or absence of the MEK inhibitor U0126 (10 μmol/l, following 30 min pre-treatment). Data represent means ± SEM, *n* = 9 wells from three independent experiments (apart from F/I, *n* = 6 wells from two independent experiments); one-way ANOVA, ***p < 0.001. (**c**) Representative traces of the [Ca^2+^]_i_ response observed in GLUTag cells following application of chylomicrons (CM; 10 μg/ml) and the positive control KCl (30 mmol/l), added as indicated by the horizontal bars. [Ca^2+^]_i_ was monitored as the ratio of fluorescence at 340 and 380 nm in individual GLUTag cells loaded with Fura-2. (**d**) Mean 340/380-nm ratio averaged over 20 s periods. Data represent means ± SEM, *n* = 44 ‘responder’ cells; Friedman test with Dunn’s correction, ***p < 0.001 vs baseline

Imaging experiments using GLUTag cells were performed as previously described [25]. Briefly, GLUTag cells were incubated with 5 μmol/l Fura-2-acetoxymethyl-ester (Fura-2-AM; Thermo Fisher Scientific, Waltham, MA, USA) and 1 mmol/l glucose in assay buffer (see below) for 15 min at 37°C and 15 min at room temperature. The Fura-2-AM solution was then replaced with assay buffer. Cells loaded with Fura-2 were continuously perfused with assay buffer with or without test reagents, and changes in intracellular Ca^2+^ levels were assessed by measuring the change in ratiometric fluorescence (excitation 340/380 nm) at wavelength above 510 nm. Experiments were performed using an Olympus IX71 inverted microscope with a 40× oil immersion objective, fitted with a monochromator (Cairn Research, Faversham, UK) and OrcaER camera (Hamamatsu, Hamamatsu City, Japan). Images were acquired every 2 s and analysed, after background subtraction, using MetaFluor software (Molecular Devices, Sunnyvale, CA, USA). Data were smoothened with a 20 s sliding average. Maximum ratios were determined at baseline (20 s prior to the test condition) and after test reagent application. Cells were included in the analysis if they responded to the positive control, 30 mmol/l KCl. Approximately 15% of cells responded to chylomicrons, and only the data from these cells (*n* = 44), termed ‘responders’ are included in Fig. 1. Summary data are presented as mean calculated increments (normalised to baseline).

### Secretion studies

Cells were washed three times with assay buffer (see below) and incubated with test reagents in assay buffer, supplemented with 0.1% fatty acid-free BSA ± 10 mmol/l glucose, for 2 h at 37°C. Test reagents were added in a random order, which differed between experiments. Supernatant and lysate samples were collected for primary cell experiments and siRNA-treated GLUTag cells. Supernatant samples only were collected for standard GLUTag secretion experiments. Samples were assessed using a total GLP-1 assay (Meso Scale Discovery, Gaithersburg, MD, USA). GLP-1 measurement was blinded. GIP was measured using a total GIP ELISA (Millipore, Billerica, MA, USA). For standard GLUTag secretion experiments, the raw GLP-1 data (in pg/ml) are presented. For primary secretion experiments and siRNA-treated GLUTag cells, hormone secretion was calculated as a percentage of total hormone content per well, to account for potential differences in EEC number between wells. GLP-1 and GIP were measured in the same samples.

### Materials

All drugs and chemicals were purchased from Sigma-Aldrich (UK), unless otherwise stated. Drugs were made up as 1000× or 10,000× stock solutions, as per the manufacturer instructions. Human chylomicrons were obtained from BioVision (Milpitas, CA, USA), and ten individual batches were used during these studies. The broad-spectrum lipase inhibitor orlistat was manufactured by Cayman Chemical (Ann Arbor, MI, USA). The FFA1 agonist AM-1638 was kindly donated by Lilly (Indianapolis, IN, USA). The FFA1 antagonist GW1100 was manufactured by Merck Chemicals (Nottingham, UK).

The assay buffer used in secretion and imaging experiments contained (in mmol/l) 138 NaCl, 4.5 KCl, 4.2 NaHCO_3_, 1.2 NaH_2_PO_4_, 2.6 CaCl_2_, 1.2 MgCl_2_ and 10 HEPES (adjusted to pH 7.4 with NaOH).

### Statistical analysis

Data are expressed as mean ± SEM unless otherwise indicated. Statistical analysis was performed using GraphPad Prism 5.00 software (San Diego, CA, USA). GLP-1 and GIP secretion data were analysed by one-way ANOVA with a post hoc Bonferroni test (*n* ≥ 3 independent experiments performed in duplicate or triplicate). For qRT-PCR data, statistical significance between the expression of the target gene in siRNA-treated vs negative control siRNA-treated GLUTag cells was assessed by an independent *t* test (*n* = 3 independent experiments performed in duplicate or triplicate). For the Ca^2+^ imaging experiments, statistical analysis was carried out using raw baseline and treatment maximum 340/380-nm ratios (*n* = 44 cells). As the data were not normally distributed and the observations were paired, they were analysed using the non-parametric Friedman test (*p* < 0.0001) with a Dunn’s post hoc test for multiple comparisons.

## Results

### Effects of chylomicrons on GLP-1 secretion and [Ca^2+^]_i_ in GLUTag cells

Chylomicrons significantly increased GLP-1 secretion from GLUTag cells (Fig. 1a; *p* < 0.05 vs control in the presence of glucose; *p* < 0.001 vs control in the presence of glucose/3-isobutyl-1-methylxanthine [IBMX]). As ERK phosphorylation has previously been linked with gut peptide release [26, 27], the ability of chylomicrons to stimulate GLP-1 secretion was tested in the presence or absence of the widely used mitogen-activated protein kinase/ERK kinase (MEK) inhibitor U0126 (Fig. 1b). Chylomicron-mediated GLP-1 secretion was significantly impaired in GLUTag cells treated with U0126 (*p* < 0.001 vs untreated cells). Glucose-mediated GLP-1 secretion also appeared to be affected by U0126, although this was not consistently observed and therefore did not reach statistical significance. On the other hand, basal secretion and GLP-1 secretion induced by forskolin/IBMX (a previously described robust stimulus) in the absence of glucose were unaffected. Due to the coupling of intracellular calcium ([Ca^2+^]_i_) elevations with gut peptide secretion, the effect of chylomicrons on [Ca^2+^]_i_ was also investigated. In a population of GLUTag cells (approximately 15%), application of chylomicrons triggered a significant rise in [Ca^2+^]_i_ (Fig. 1c, d).

### Dependence of chylomicron-mediated GLP-1 secretion on lipoprotein lipase in GLUTag cells

**Fig. 2:**
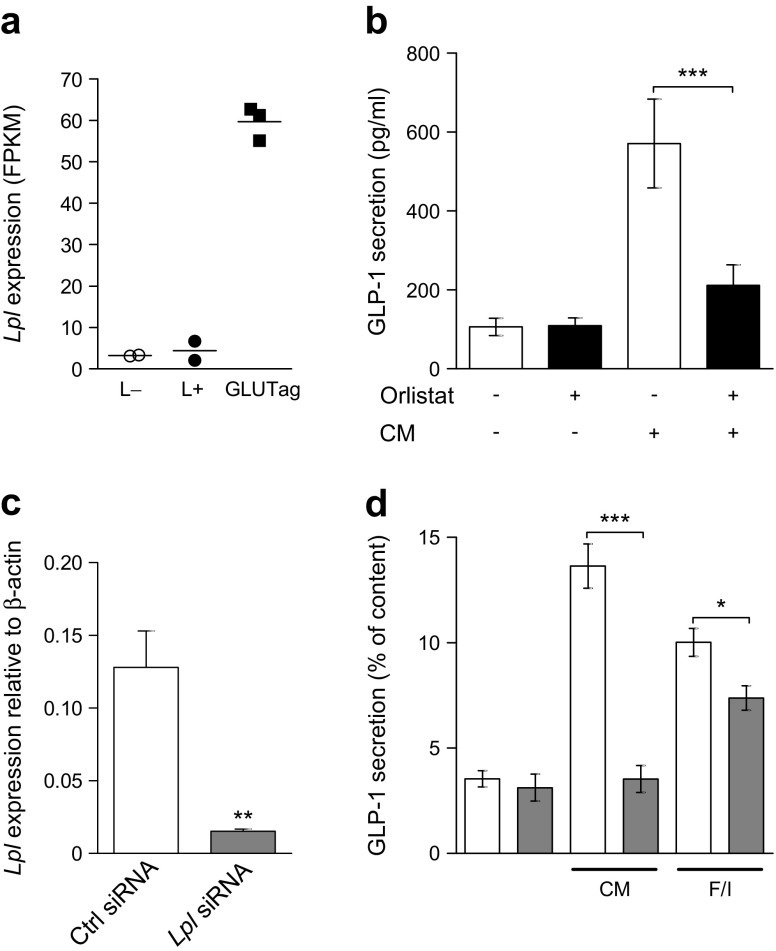
Chylomicron-mediated GLP-1 release in GLUTag cells is dependent on LPL. (**a**) *Lpl* expression was examined by RNA sequencing FACS-sorted primary L cells (GLU-Venus-positive, L+) and negative cells (GLU-Venus-negative, L−) collected in parallel from murine duodenum and GLUTag cells. FPKM, fragments per kilobase per million reads. (**b**) GLP-1 secretion from GLUTag cells treated with chylomicrons (CM; 10 μg/ml) in the presence or absence of orlistat (1 μg/ml, following 30 min pre-treatment). Data represent means ± SEM, *n* = 9 wells from three independent experiments; one-way ANOVA, ***p < 0.001. (**c**) GLUTag cells were transfected with 30 nmol/l *Lpl* siRNA or negative control (Ctrl) siRNA, and knockdown was validated by qRT-PCR. Data are presented as means ± SEM, *n* = 5–6 from three independent experiments; unpaired *t* test, **p < 0.01. (**d**) GLP-1 secretion from negative control (white bars) or *Lpl* siRNA (grey bars) transfected GLUTag cells treated with chylomicrons (CM; 10 μg/ml) or forskolin/IBMX (F/I; 10 μmol/l each) in the presence of glucose (10 mmol/l). Data represent means ± SEM, *n* = 8–9 wells from three independent experiments; one-way ANOVA, *p < 0.05, ***p < 0.001

To further dissect the mechanisms underlying chylomicron-mediated GLP-1 secretion, genes of interest were identified from an RNA-Seq database derived from duodenal GLU-Venus-positive ‘L cells’ or negative control ‘non-L cells’, as well as GLUTag cells. One gene that was identified as being expressed in primary duodenal L cells and also highly expressed in GLUTag cells was *Lpl* (Fig. 2a).

Treatment of GLUTag cells with the broad-spectrum lipase inhibitor orlistat (tetrahydrolipstatin) [28] significantly blunted chylomicron-induced GLP-1 secretion (Fig. 2b; *p* < 0.001 vs untreated cells). To support these results, GLUTag cells were also transfected with *Lpl* siRNA, or negative siRNA as a control. Post-transfection (48 h), a significant knockdown of mRNA expression (approximately 90% reduction) was achieved (*p* < 0.01 vs negative siRNA), as determined by qRT-PCR (Fig. 2c). Secretion experiments using these transfected cells demonstrated that GLP-1 release in response to chylomicrons was practically abolished when *Lpl* expression was knocked down (Fig. 2d). While there was a significant difference in GLP-1 release following incubation with the positive control forskolin/IBMX, this difference was no longer significant when expressed as ‘relative to basal’, whereas the inhibition of chylomicron-stimulated secretion remained significant (*p* < 0.001; data not shown).

### Role of FFA1 in chylomicron-induced GLP-1 secretion in GLUTag cells

**Fig. 3:**
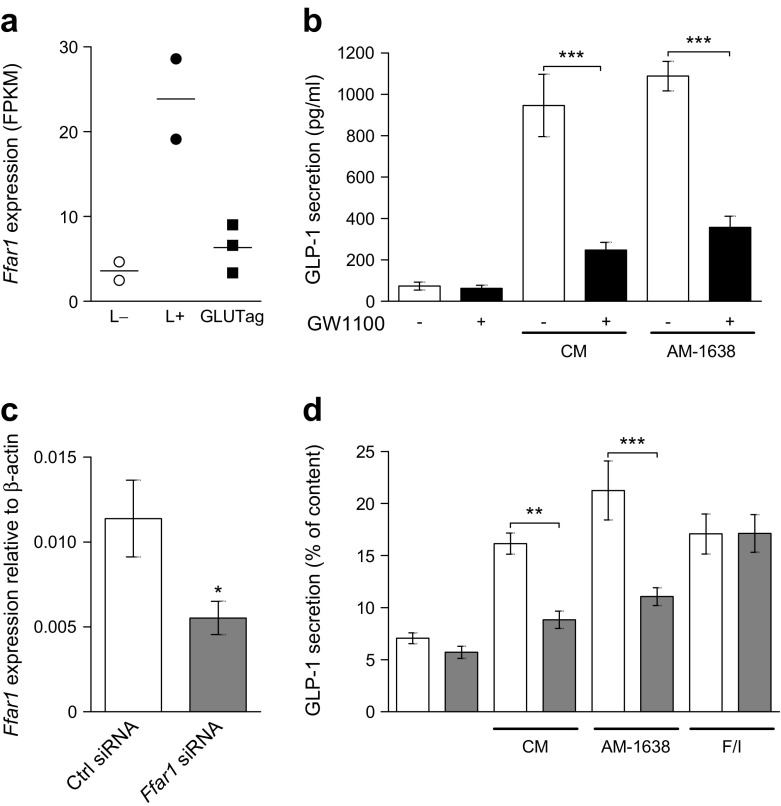
Chylomicrons stimulate GLP-1 release via FFA1 in GLUTag cells. (**a**) FFA1 (*Ffar1*) expression was examined by RNA sequencing FACS-sorted primary L cells (GLU-Venus-positive, L+) and negative cells (GLU-Venus-negative, L−) collected in parallel from murine duodenum and GLUTag cells. FPKM, fragments per kilobase per million reads. (**b**) GLP-1 secretion from GLUTag cells treated with chylomicrons (CM; 10 μg/ml) or the selective FFA1 agonist AM-1638 (1 μmol/l) in the presence or absence of the FFA1 antagonist GW1100 (1 μmol/l, with 30 min pre-treatment). Data represent means ± SEM, *n* = 9 wells from three independent experiments; one-way ANOVA, ***p < 0.001. (**c**) GLUTag cells were transfected with 30 nmol/l *Ffar1* siRNA or negative control (Ctrl) siRNA, and knockdown was validated by qRT-PCR. Data are presented as means ± SEM, *n* = 7 from three independent experiments; unpaired *t* test, *p < 0.05. (**d**) GLP-1 secretion from negative control (white bars) or *Ffar1* siRNA (grey bars) transfected GLUTag cells treated with chylomicrons (CM; 10 μg/ml), AM-1638 (1 μmol/l) or forskolin/IBMX (F/I; 10 μmol/l each) in the presence of glucose (10 mmol/l). Data represent means ± SEM, *n* = 9 wells from three independent experiments; one-way ANOVA, **p < 0.01, ***p < 0.001

As *Ffar1* is known to be expressed by L cells and GLUTag cells (Fig. 3a), we examined its role in potentially mediating the effect of long-chain NEFAs, generated by chylomicron hydrolysis, using the FFA1 antagonist GW1100. The synthetic FFA1 selective agonist AM-1638 led to a significant increase in GLP-1 secretion (*p* < 0.001 vs control) and was thus used as a positive control. In the presence of GW1100, GLP-1 secretion in response to both AM-1638 and chylomicrons was significantly inhibited in GLUTag cells (Fig. 3b).

To support these findings, GLUTag cells were also transfected with *Ffar1* siRNA or negative control siRNA. Following transfection (48 h), *Ffar1* expression was reduced by approximately 50% (Fig. 3c, *p* < 0.05 vs negative siRNA). GLP-1 secretion in response to both AM-1638 and chylomicrons was significantly inhibited in GLUTag cells transfected with *Ffar1* siRNA compared with negative siRNA (Fig. 3d; *p* < 0.001 and *p* < 0.01, respectively). Basal secretion and the response to forskolin/IBMX were unaltered by transfection with *Ffar1* siRNA.

### Effects of chylomicrons on GLP-1 and GIP secretion from human and murine primary intestinal cultures

**Fig. 4:**
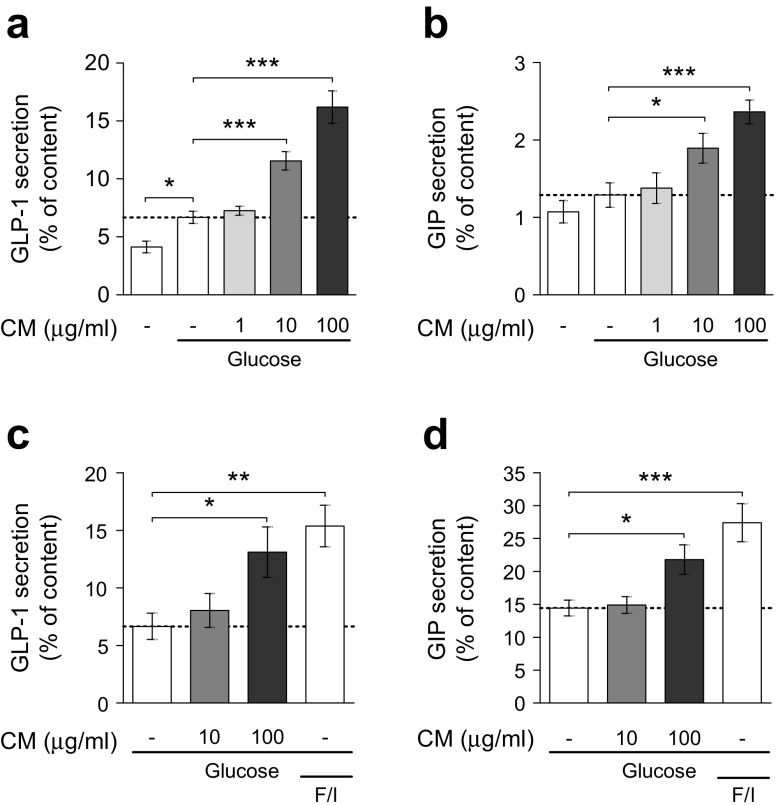
Chylomicrons stimulate GLP-1 and GIP secretion from human and murine primary duodenal cultures. (**a**) GLP-1 and (**b**) GIP secretion from murine duodenal cultures treated with chylomicrons (CM) in the presence of glucose (10 mmol/l). Data represent means ± SEM, *n* = 12 wells (except for 100 μg/ml, *n* = 9 wells) from four independent experiments; one-way ANOVA, *p < 0.05, ***p < 0.001. (**c**) GLP-1 and (**d**) GIP secretion from human duodenal cultures treated with chylomicrons or the positive control forskolin/IBMX (F/I; 10 μmol/l each) in the presence of glucose (10 mmol/l). Data represent means ± SEM, *n* = 9–10 wells (except for 10 μg/ml, *n* = 6 wells) from three independent experiments; one-way ANOVA, *p < 0.05, **p < 0.01, ***p < 0.001

To determine whether the stimulatory effect of chylomicrons was maintained in primary cultures, GLP-1 and GIP secretion was measured following incubation of duodenal cultures, of both murine and human origin, with chylomicrons. Chylomicrons at physiological concentrations of 10 and 100 μg/ml [29] significantly stimulated GLP-1 and GIP secretion in murine duodenal cultures, in a dose-dependent fashion (Fig. 4a, b). Furthermore, chylomicrons also led to a significant increase in both GLP-1 and GIP secretion from human duodenal cultures (Fig. 4c, d).

### Mechanisms underlying chylomicron-mediated GLP-1 and GIP secretion from primary EECs

**Fig. 5:**
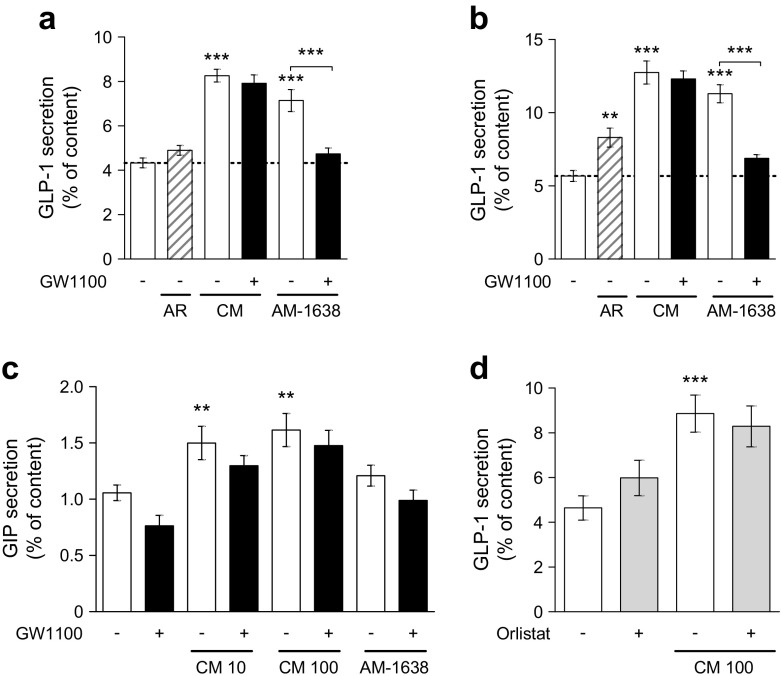
FFA1 does not play a role in chylomicron-mediated GLP-1 and GIP secretion in murine primary cultures. (**a**) GLP-1 secretion from L cell-specific *Gpr119* knockout (*Cre*-positive/*Gpr119* ^flox^) and (**b**) wild-type-like (*Cre*-negative/*Gpr119* ^flox^) murine duodenal cultures treated with the GPR119 agonist AR231453 (AR; 100 nmol/l), chylomicrons (CM; 100 μg/ml) or the FFA1 agonist AM-1638 (1 μmol/l), in the presence or absence of the FFA1 antagonist GW1100 (5 μmol/l, 30 min pre-treatment). Data represent means ± SEM, *n* = 9 wells from three independent experiments; one-way ANOVA, **p < 0.01, ***p < 0.001 vs glucose (10 mmol/l) control. (**c**) GIP secretion from murine duodenal cultures treated with chylomicrons (CM; 10 or 100 μg/ml) or AM-1638 (1 μmol/l), in the presence or absence of GW1100 (5 μmol/l, 30 min pre-treatment). Data represent means ± SEM, *n* = 11–21 wells from 4–7 independent experiments; one-way ANOVA, **p < 0.01 vs glucose (10 mmol/l) control. (**d**) GPL-1 secretion from murine duodenal cultures treated with chylomicrons (CM; 100 μg/ml), in the presence or absence of orlistat (10 μg/ml, 30 min pre-treatment). Data represent means ± SEM, *n* = 11–12 wells from four independent experiments; one-way ANOVA, ***p < 0.001 vs glucose (10 mmol/l) control

The relative contributions of FFA1 and GPR119 in chylomicron-induced GLP-1 secretion were investigated using duodenal cultures from conditional *Gpr119* knockout mice lacking GPR119 in proglucagon-expressing cells (*Cre*-positive/*Gpr119* ^flox^), and the FFA1 antagonist GW1100. The absence of GPR119 in L cells did not impair GLP-1 release in response to chylomicrons (Fig. 5a). However, it did prevent GLP-1 secretion in response to the GPR119 agonist AR231453 (Fig. 5a), which in the wild-type-like *Cre*-negative*/Gpr119* ^fl^ cultures significantly stimulated GLP-1 secretion (Fig. 5b). As anticipated, the FFA1 agonist AM-1638 significantly stimulated GLP-1 secretion in the primary cultures, an effect that was inhibited by the FFA1 antagonist GW1100 (Fig. 5a, b). As GPR119 and FFA1 have been shown to act synergistically [11, 14], we also tested the ability of chylomicrons to stimulate GLP-1 secretion in a setting where both pathways were inhibited, i.e. in L cell-specific *Gpr119* knockout (*Cre*-positive/*Gpr119* ^flox^) cultures treated with GW1100. In the absence of both GPR119 and FFA1 signalling, chylomicrons were still able to significantly increase GLP-1 secretion (Fig. 5a).

To assess the role of FFA1 in mediating the stimulatory effect of chylomicrons on GIP secretion, primary murine duodenal cultures were treated with chylomicrons (10 and 100 μg/ml) and AM-1638 in the presence or absence of GW1100. As with GLP-1, the inhibition of FFA1 had no effect on chylomicron-mediated GIP secretion from duodenal cultures (Fig. 5c). Moreover, in our hands, AM-1638 did not significantly stimulate GIP secretion (Fig. 5c).

Furthermore, treatment of duodenal cultures with the lipoprotein lipase (LPL) inhibitor orlistat had no effect on chylomicron-stimulated GLP-1 secretion (Fig. 5d).

## Discussion

This study has demonstrated that chylomicrons, at physiological concentrations [29], stimulate incretin hormone secretion from GLUTag cells as well as from primary duodenal cultures of both human and murine origin. In GLUTag cells, the molecular pathway was found to involve LPL-mediated lipolysis, leading to the release of lipid species that activated FFA1 and elevated intracellular calcium.

The importance of LPL in GLUTag cells was independently implicated by pharmacological inhibition and genetic manipulation, as chylomicron-triggered GLP-1 secretion was largely abolished following incubation with the broad-spectrum lipase inhibitor orlistat [28] or siRNA-mediated knockdown of *Lpl* mRNA expression. LPL catalyses the hydrolysis of chylomicron triacylglycerols to produce LCFAs and monoacylglycerols, which are both well-known stimuli of GLP-1 secretion acting via FFA1 and GPR119, respectively. In GLUTag cells, both FFA1 antagonism using GW1100 [30] and siRNA-mediated *Ffar1* knockdown reduced GLP-1 secretion in response to chylomicrons, and, consistent with the known G_q_-coupling of FFA1, chylomicrons triggered an increase in intracellular Ca^2+^. The MEK inhibitor U0126 also significantly inhibited chylomicron-triggered GLP-1 secretion, suggesting an involvement of MEK–ERK signalling in the secretory pathway downstream of chylomicrons. Although ERK signalling has been implicated in the GLP-1 secretory response to a variety of stimuli [26, 27], how ERK couples to secretion remains to be determined. Taken together, these data suggest that in GLUTag cell cultures, chylomicrons are hydrolysed by LPL to liberate LCFAs, which subsequently activate FFA1 to trigger GLP-1 secretion. It is unclear whether LPL adheres to the surface of GLUTag cells, potentially via heparan sulphate proteoglycans, or whether it is secreted into the medium, or indeed both.

Chylomicrons significantly stimulated both GLP-1 and GIP secretion in human and murine duodenal cultures, contrasting with the findings of Chandra et al. [31], who reported that chylomicrons at the same concentration (100 μg/ml) stimulated cholecystokinin (CCK) secretion only in the additional presence of the fatty acid C12 (100 μmol/l), pointing towards possible differences in the stimulus detection mechanisms for CCK and incretin secretion. In primary intestinal cultures, however, orlistat did not inhibit chylomicron-mediated GLP-1 secretion, and no impairment of chylomicron-triggered GLP-1 secretion was observed when the GPR119 and FFA1 pathways were inactivated. Although we cannot rule out the possibility that orlistat was inactivated during the incubation period [32], the additional lack of evidence for involvement of GPR119 or FFA1 suggests that LPL-dependent hydrolysis of triacylglycerols in chylomicrons does not underlie the stimulation of GLP-1 and GIP secretion observed in primary cultures. This is not the first demonstration of nutrients being sensed via distinct mechanisms in GLUTag cells and primary L cells (e.g. the role of GPRC6A in GLUTag cells but not native L cells [33, 34]). As GLUTag cells were originally derived from an intestinal tumour, it is conceivable that their high expression of LPL may reflect a cancerous phenotype. LPL has been detected in a number of tumour types and has been shown to be critical in enabling cancer cells to acquire NEFAs from culture medium [35].

Given that lipolysis does not appear to be required for chylomicron-dependent GLP-1 secretion from primary intestinal cultures, it is possible that this effect is mediated by a lipoprotein or scavenger receptor expressed by primary L cells. Chandra et al. [31] found that CCK secretion in response to the combination of HDL and C12 was significantly inhibited in I cells lacking the ILDR1 receptor. However, CCK release in response to the combination of chylomicrons and C12 was not significantly reduced by ILDR1 deficiency, suggesting the involvement of an additional pathway for chylomicron sensing. This might involve the detection of molecules on the chylomicron surface, or of other lipid species released from the chylomicron core. Interestingly, apolipoprotein A-IV, present on the surface of chylomicrons, has been implicated in the suppression of gastric motility and food intake, via an increase in CCK secretion [36, 37], as well as in glucose homeostasis via an increase in insulin secretion [38]. However, a specific receptor for apolipoprotein A-IV has yet to be identified, and purified human apolipoprotein A-IV or apolipoprotein A-I failed to stimulate GLP-1 secretion in primary murine epithelial cultures (see electronic supplementary material [ESM] Fig. 1). As the preservation of the apical/basolateral polarisation and limited access to only the basolateral compartment are compromised in primary epithelial cultures, we cannot exclude that the observed stimulation by chylomicrons is a result of artificial access to apically located receptors; however, we can exclude GPR119 and FFA1 as possible mediators under these culture conditions, as discussed above. By contrast, we cannot exclude apolipoprotein A-I or A-IV as possible mediators, as the results of the pilot experiments might simply reflect conformational changes due to the purification of the apolipoproteins.

The release of gut peptides in response to chylomicrons may have physiological relevance beyond the classical slowing of gastrointestinal motility and increase in satiety. In addition to producing GLP-1, primary L cells also produce GLP-2, from the processing of the proglucagon precursor. GLP-2 is best known for its role in stimulating intestinal growth and promoting nutrient absorption [39, 40], including fat absorption via an upregulation of CD36/fatty acid translocase [41]. Intriguingly, there is emerging evidence to suggest that GLP-2 also promotes the release of preformed chylomicrons from enterocytes in humans and animal models [42, 43], an effect that under normal physiological conditions is thought to predominate over the opposing action of GLP-1 [44]. The mechanisms underlying this effect are currently unclear and are presumably indirect, given that neither the GLP-1 nor the GLP-2 receptor is thought to be expressed by enterocytes.

The idea that chylomicron formation is important for EEC detection of ingested lipids was suggested previously by in vivo experiments in rats, which demonstrated that co-administration of an intraduodenal lipid emulsion with Pluronic L-81, an inhibitor of chylomicron formation [45, 46], significantly impaired GLP-1 secretion and largely abolished GIP secretion [18]. Other in vivo studies have shown that oral lipid-triggered GLP-1 and GIP secretion is strongly dependent on FFA1 and GPR119, and that FFA1 on L cells responds primarily to ligands delivered from the basolateral direction [13, 14, 16]. Our results from murine and human cultures demonstrating an effect of chylomicrons on gut hormone secretion and the molecular pathway highlighted in GLUTag cells could explain a wealth of these in vivo findings.

**Fig. 6:**
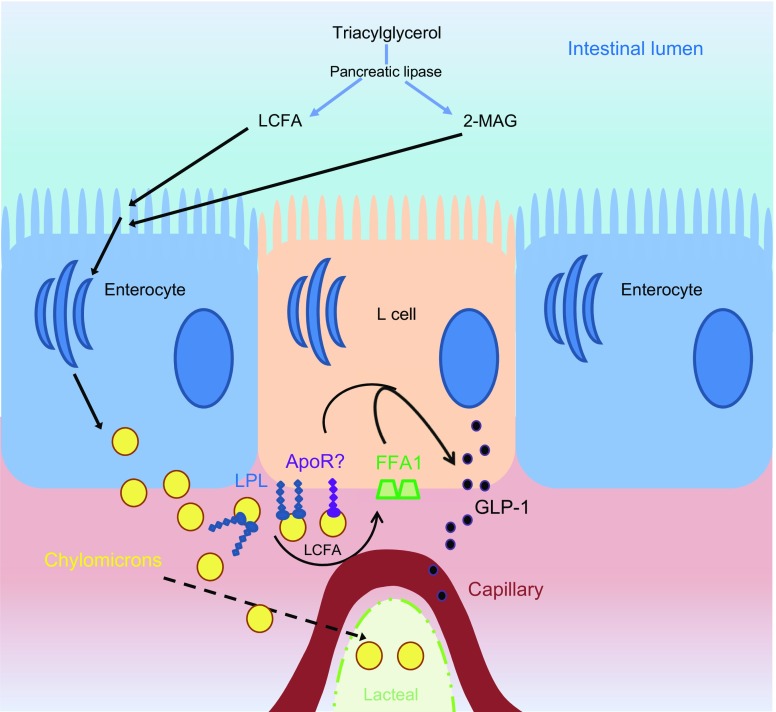
Schematic of proposed revised model of intestinal fat sensing. Rather than stimulating GLP-1 secretion through apically located long-chain NEFA- and/or monoacylglycerol-sensing receptors, we propose the re-esterification and secretion of chylomicrons from enterocytes to be an essential step in lipid-sensing of intestinal L cells. LPL expressed either by L cells or other cells within the villus core sufficiently hydrolyses triacylglycerols to stimulate basolaterally located FFA1. The results in primary mixed epithelial cultures suggest additional sensing mechanisms independent of triacylglycerol hydrolysis through yet to be identified receptors (‘ApoR’). 2-MAG, 2-monoacylglycerol

This model (Fig. 6) involves a chylomicron-dependent step for delivery of lipids to the basolateral compartment of the intestinal epithelium, followed by the local lipase-dependent production of active lipid species such as LCFAs and monoacylglycerols, which target GPCRs such as FFA1 and GPR119 on the basolateral membrane of EECs and thereby stimulate gut peptide secretion. Although we could not demonstrate the validity of this molecular pathway in the primary duodenal culture model, we do not believe this rules out the possibility that the pathway plays an important role in the intact gut, where other non-epithelial cells lost in the cultures might contribute to partial chylomicron lipolysis. Therefore, further work is warranted to establish the importance of local lipase-dependent LCFA and monoacylglycerol delivery from chylomicrons for the stimulation of gut hormone release by ingested lipids.

## Electronic supplementary material

ESM Fig. 1 (PDF 70 kb)

## Abbreviations

[Ca^2+^]_i_: Intracellular calcium concentration
CCK: Cholecystokinin
EEC: Enteroendocrine cell
ERK: Extracellular signal-regulated kinase
FFA1/4: Free fatty acid receptor 1/4
Fura-2-AM: Fura-2-acetoxymethyl ester
GIP: Glucose-dependent insulinotropic peptide
GLP: Glucagon-like peptide
GPCR: G-protein-coupled receptor
GPR119: G-protein coupled receptor 119
IBMX: 3-Isobutyl-1-methylxanthine
ILDR1: Immunoglobulin-like domain containing receptor 1
LCFA: Long-chain fatty acid
LPL: Lipoprotein lipase
MEK: Mitogen-activated protein kinase kinase
qRT-PCR: Quantitative reverse transcription PCR
siRNA: Small (short) interfering RNA
