# Supplementary material for: Chylomicrons stimulate incretin secretion in mouse and human cells
Source: Diabetologia. 2017 Sep 2;60(12):2475–85. doi: 10.1007/s00125-017-4420-2 (PMC5850988; doi:10.1007/s00125-017-4420-2)
Supplement: Supplementary file 1 — (PDF 70 kb) [file 125_2017_4420_MOESM1_ESM.pdf]

ESM Fig. 1

a

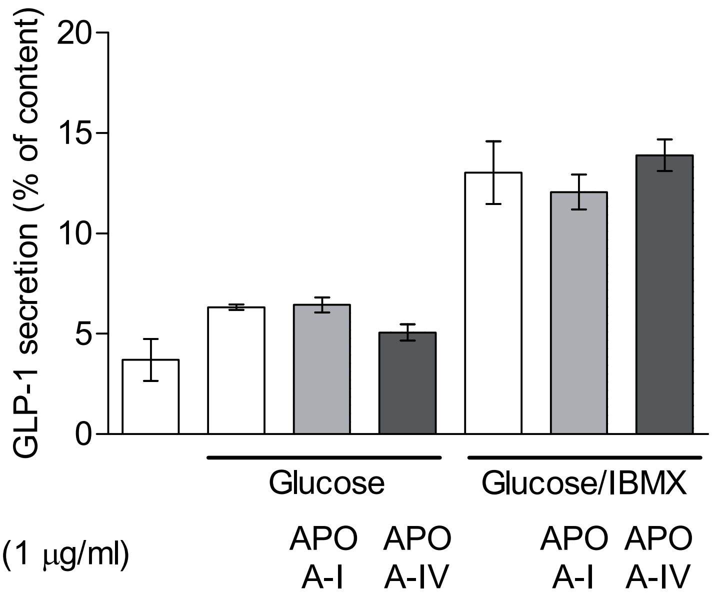

b

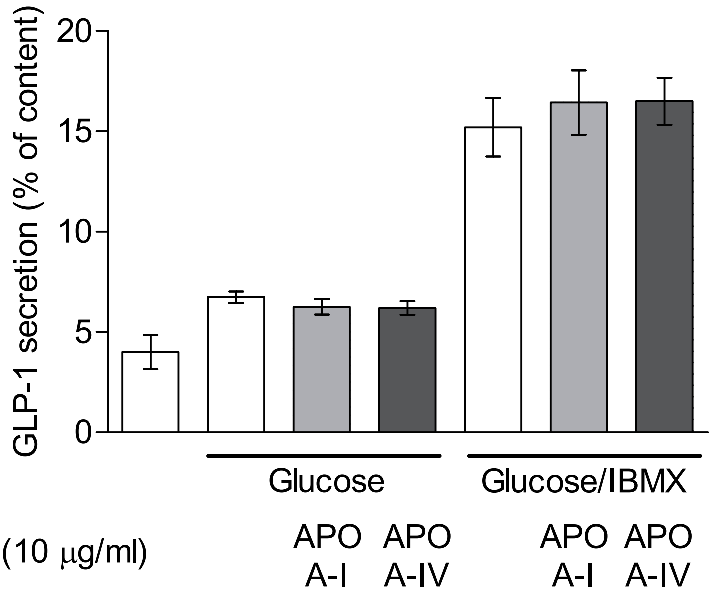

ESM Fig. 1 Effect of apolipoprotein A-I and A-IV on GLP-1 secretion.
GLP-1 secretion from primary murine duodenal cultures in response to purified apolipoprotein A-I (APOA-I) and apolipoprotein A-IV (APOA-IV) at concentrations of (a) 1μg/ml and (b) 10 μg/ml in the presence of 10 mmol/l glucose ± 100μmol/l IBMX. Data represent means ± SEM (n=3 wells). APOA-I, purified from human plasma, was purchased from CellBiolabs (San Diego, CA, USA) as 1 mg/ml stock. APOA-IV, purified from human plasma, was purchased from BioVision (Milpitas, CA, USA) as 0.4 mg/ml stock. Both were diluted into assay-buffer based (see Methods in the main paper) test solution on the day of the experiment
